# Supplementary material for: One-year experience with latanoprostene bunod ophthalmic solution 0.024% in clinical practice: A retrospective observational study
Source: PLoS One. 2024 Aug 22;19(8):e0307132. doi: 10.1371/journal.pone.0307132 (PMC11341023; doi:10.1371/journal.pone.0307132)
Supplement: S1 Table — (DOCX) [file pone.0307132.s002.docx]

**S1-Table**. Mean additional intraocular pressure (IOP) reduction after switched to LBN in patients with primary open angle glaucoma (POAG) or normal tension glaucoma (NTG).

|  | | | |  |
| --- | --- | --- | --- | --- |
|  | POAG  n=22 | NTG  n=59 | P value |  |
| IOP reduction  1 M (mmHg) | 2.1±2.2 | 0.8±1.9 | 0.03 |  |
| 3 M (mmHg) | 2.3±2.8 | 0.9±1.8 | 0.02 |  |
| 6 M (mmHg)  12M (mmHg) | 2.1±2.9  2.1±3.2 | 0.9±2.2  1.3±2.0 | 0.1  0.2 |  |

PGA=Prostaglandin analogue; M=month; LBN=latanoprostene bunod
