# Supplementary material for: One-year experience with latanoprostene bunod ophthalmic solution 0.024% in clinical practice: A retrospective observational study
Source: PLoS One. 2024 Aug 22;19(8):e0307132. doi: 10.1371/journal.pone.0307132 (PMC11341023; doi:10.1371/journal.pone.0307132)
Supplement: S2 Table — (DOCX) [file pone.0307132.s003.docx]

**S2-Table**. Mean additional intraocular pressure (IOP) reduction in patients treated with tafluprost or travoprost before switched to LBN

|  | | | |  |
| --- | --- | --- | --- | --- |
| PGA | Tafluprost (n=46) | Travoprost  (n=31) | P value |  |
| IOP reduction  1 M (mmHg) | 1.6±1.9 | 0.1±1.6 | 0.001 |  |
| 3 M (mmHg) | 1.4±2.1 | 0.6±1.7 | 0.07 |  |
| 6 M (mmHg)  12M (mmHg) | 1.3±2.2  1.8±2.3 | 0.6±2.3  0.5±1.7 | 0.2  0.02 |  |

PGA=Prostaglandin analogue; M=month
