# Supplementary material for: One-year experience with latanoprostene bunod ophthalmic solution 0.024% in clinical practice: A retrospective observational study
Source: PLoS One. 2024 Aug 22;19(8):e0307132. doi: 10.1371/journal.pone.0307132 (PMC11341023; doi:10.1371/journal.pone.0307132)
Supplement: Attachment — Submitted filename: Response to reviewers.docx [file pone.0307132.s005.docx]

Response to reviewers:

Dear PLOS ONE editor and reviewers:

Thank you for having us an opportunity to revise our manuscript. We are so appreciated for your kindness and help.

Journal Requirements:

1. We already correct the style and ensure that our manuscript meets PLOS ONE's style requirements
2. We have included captions for our Supporting Information files at the end of our manuscript, and update in-text citations to match accordingly.
3. We have reviewed our reference list to ensure that it is complete and correct.

Editor Comments:
 Thank you for giving us many useful suggestions about the article. We grouped the adverse effects into tolerable and intolerable side effects, and compare the side effects reported in literature for both latanoprostene bunod, as well as prostaglandin analogues used alone.

Reviewers' comments:

1. Real-world observation studies, such as medical chart reviews, can provide clinicians with timely insight on the potential effectiveness of new treatments in patient populations encountered in clinical practice but not specifically included in clinical trials. The sample size 78 patients included initially was no based on power considerations for testing any hypothesis and was assumed to provide data to study the clinical effect and safety profile of LBN 0.024% over 12 months using descriptive statistics in the real world practice.
2. The differences of IOP reduction between baseline IOP, PGA IOP and IOP with LBN 0.024% treatment at 1-, 3-, 6- and 12-months were calculated using linear mixed-effects models, with fixed effect terms for treatment and period, and a within-participant random intercept. All statistical analyses were performed using SPSS (version 26.0; SPSS Inc, Chicago, IL, USA)
3. 4. Typographical or grammatical errors have be corrected at revision.

Reviewer 1:

(1) Intraocular pressure measurements used non-contact tonometer (TONOREF III, NIDEK, Japan) and rechecked with iCare tonometer (TA01i). If two measurements are different, IOP data from iCare tonometer was recorded.
(2) Approximately 40% of patients have intolerable ocular adverse effects, mostly moderate to severe conjunctival hyperemia and instillation site pain, so they dropped out at 1 month of LBN use and changed medication. The dropped out rate was higher than reported in the prior study. There are some possible causes: First, differences in the ethnic populations and different study design may have contributed to the greater dropped out rate observed in the present study. Second, the higher dropped out rate in the present study may be due to direct switch from PG analog (travoprost and tafluprost) to LBN. Washout period may be important to increase the adherence. Third, mean age of patients in the present study (51.3 years) were younger than in the pooled analysis study and Jupiter study (64.9 and 62.5 years). Younger patients may have higher possibility to intolerant the ocular adverse effects due to the conjunctival hyperemia and ocular irritation.
(3) Future prospective studies with larger samples and longer follow-up periods are warranted. The high dropped out rate due to adverse effects warrant further investigations into patient-reported outcomes and quality of life measures.

Reviewer 2:

(1) We have rewrote the manuscript with clear and correct sentences.
(2) Purpose: We evaluated the IOP-lowering efficacy and safety of latanoprostene bunod (LBN) ophthalmic solution 0.024% (Vyzulta®), the first topical nitric oxide-donating prostaglandin analog (PGA), in clinical practice.
(3) Methods in Abstract: All included patients received LBN 0.024% as a replacement for a PGA, with examinations at 1-, 3- , 6-and 12-months follow-up.
(4) Baseline IOP: patients without any anti-glaucoma drugs

PGA-IOP: IOP with prostaglandin analog treatment

Addition mean IOP reduction: IOP difference between PGA-IOP and IOP after switched to LBN

(5) Among seventy-eight included patients, 47 patients (81 eyes, 60 %) with open-angle glaucoma (OAG) remained on LBN treatment throughout 12-months follow-up. 31 patients (40%) dropped out for intolerable adverse effects after 1 month use of LBN.
(6) **S1-Table**. Mean additional intraocular pressure (IOP) reduction after switched to LBN in patients with primary open angle glaucoma (POAG) or normal tension glaucoma (NTG).

**S2-Table**. Mean additional intraocular pressure (IOP) reduction in patients treated with tafluprost or travoprost before switched to LBN

(7) 31 patients (40%) dropped out for intolerable adverse effects

6. PLOS authors have the option to publish the peer review history of their article.

YES!
